# Functional genomics and the future of iPSCs in disease modeling

**DOI:** 10.1016/j.stemcr.2022.03.019

**Published:** 2022-04-28

**Authors:** Imogen R. Brooks, Cristina M. Garrone, Caoimhe Kerins, Cher Shen Kiar, Sofia Syntaka, Jessie Z. Xu, Francesca M. Spagnoli, Fiona M. Watt

**Affiliations:** 1 St John’s Institute of Dermatology, King’s College London, London, SE1 9RT, UK; 2 Centre for Gene Therapy and Regenerative Medicine, King’s College London, London, SE1 9RT, UK; 3 Centre for Craniofacial and Regenerative Biology, King’s College London, London, SE1 9RT, UK; 4 Peter Gorer Department of Immunobiology, King’s College London, London, SE1 9RT, UK; 5 Directors' Research Unit, European Molecular Biology Laboratory, Heidelberg, Germany

**Keywords:** hiPSCs, functional genomics, genetic variants, disease modeling, drug screening

## Abstract

Induced pluripotent stem cells (iPSCs) are valuable in disease modeling because of their potential to expand and differentiate into virtually any cell type and recapitulate key aspects of human biology. Functional genomics are genome-wide studies that aim to discover genotype-phenotype relationships, thereby revealing the impact of human genetic diversity on normal and pathophysiology. In this review, we make the case that human iPSCs (hiPSCs) are a powerful tool for functional genomics, since they provide an *in vitro* platform for the study of population genetics. We describe cutting-edge tools and strategies now available to researchers, including multi-omics technologies, advances in hiPSC culture techniques, and innovations in drug development. Functional genomics approaches based on hiPSCs hold great promise for advancing drug discovery, disease etiology, and the impact of genetic variation on human biology.

## Graphical abstract

**Figure:**
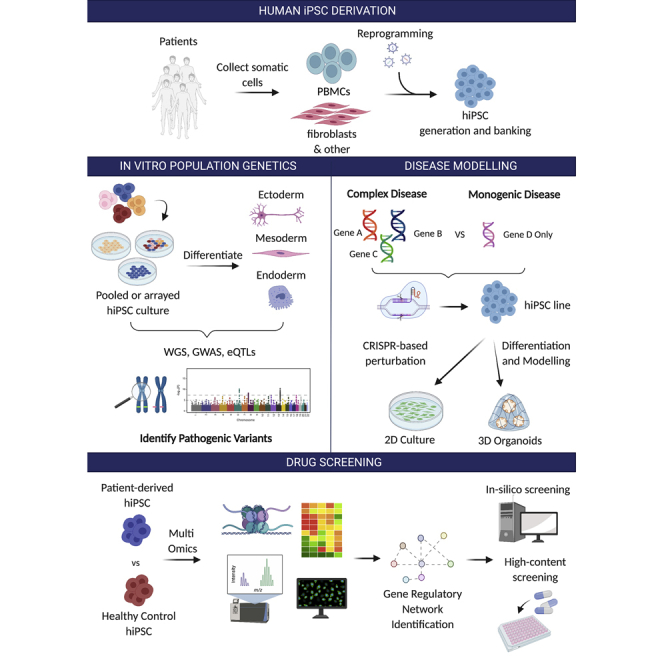

## Introduction

Advances in understanding disease biology and drug development depend on the availability of reproducible and accurate disease models. The alarmingly high failure rates of drugs in clinical trials, notably in the case of Alzheimer’s disease (AD) (Oxford et al., 2020), are a sign of the inability of current pre-clinical models to fully recapitulate disease biology and predict clinical outcomes.

To date, it has been standard practice to employ animal models to study how diseases may manifest and progress in humans. However, the lack of congruence between animal models and human diseases (Van Norman, 2019) has led to failures in translation of numerous pre-clinical and clinical trial results. Besides animal models, *in vitro* models of disease have been mostly based on two-dimensional (2D) culture of immortalized human cancer cell lines, such as HeLa cells (Augustine et al., 2021; Wolinetz and Collins, 2020). While these cell lines have proved very useful in the past, it is important for future development of disease modeling to obtain multiple cell types from a diverse range of patients. Indeed, by profiling and modeling a disease in a range of patient cells, one can begin to understand the impact of genetic background on disease. Primary cells from patients would be an ideal solution to address this gap. The lack of availability of primary cells from patients, particularly in the case of neuronal, heart, and pancreatic tissues, due to their limited *in vitro* proliferative capacity, is a major problem. However, induced pluripotent stem cell (iPSC) technologies (Takahashi and Yamanaka, 2006) allow the establishment of patient-derived cells carrying all the genetic alterations underlying a particular disease.

Takahashi and Yamanaka (2006) reprogrammed somatic cells into iPSCs. Since then, many protocols have been established to differentiate hiPSCs into a range of cell types, including cardiomyocytes (Lemoine et al., 2017), hematopoietic cells (Jeong et al., 2020), neurons (Shi et al., 2012), glia cells (Canals et al., 2018), and pancreatic beta cells (Pagliuca et al., 2014). These have been used for *in vitro* disease modeling (Rowe and Daley, 2019), drug screening (Rowe and Daley, 2019; Silva and Haggarty, 2020), and autologous transplantation (Barker et al., 2017; Mandai et al., 2017). hiPSCs are particularly well positioned to generate suitable *in vitro* pre-clinical disease models as they (1) recapitulate disease biology under physiological conditions, (2) possess high proliferative capacity, (3) have the potential to yield multiple cell types, and (4) retain patient genetic signatures. Furthermore, bioengineering methods, such as microfluidics and synthetic materials, have been able to mimic developmental cues, facilitating autonomous cellular organization of hiPSCs into complex three-dimensional (3D) organoids (Garreta et al., 2021). The immense potential of hiPSC-based disease models in advancing disease biology and regenerative medicine was recognized very early on, and has led to a few extensive efforts, such as the Human Induced Pluripotent Stem Cell Initiative (HipSci), to bank well-characterized hiPSC lines as a resource for basic and translational research (Leha et al., 2016). Together, hiPSC-based models offer a route to recapitulate human disease biology and create *in vitro* patient-specific platforms for drug development (Leha et al., 2016).

**Figure 1:**
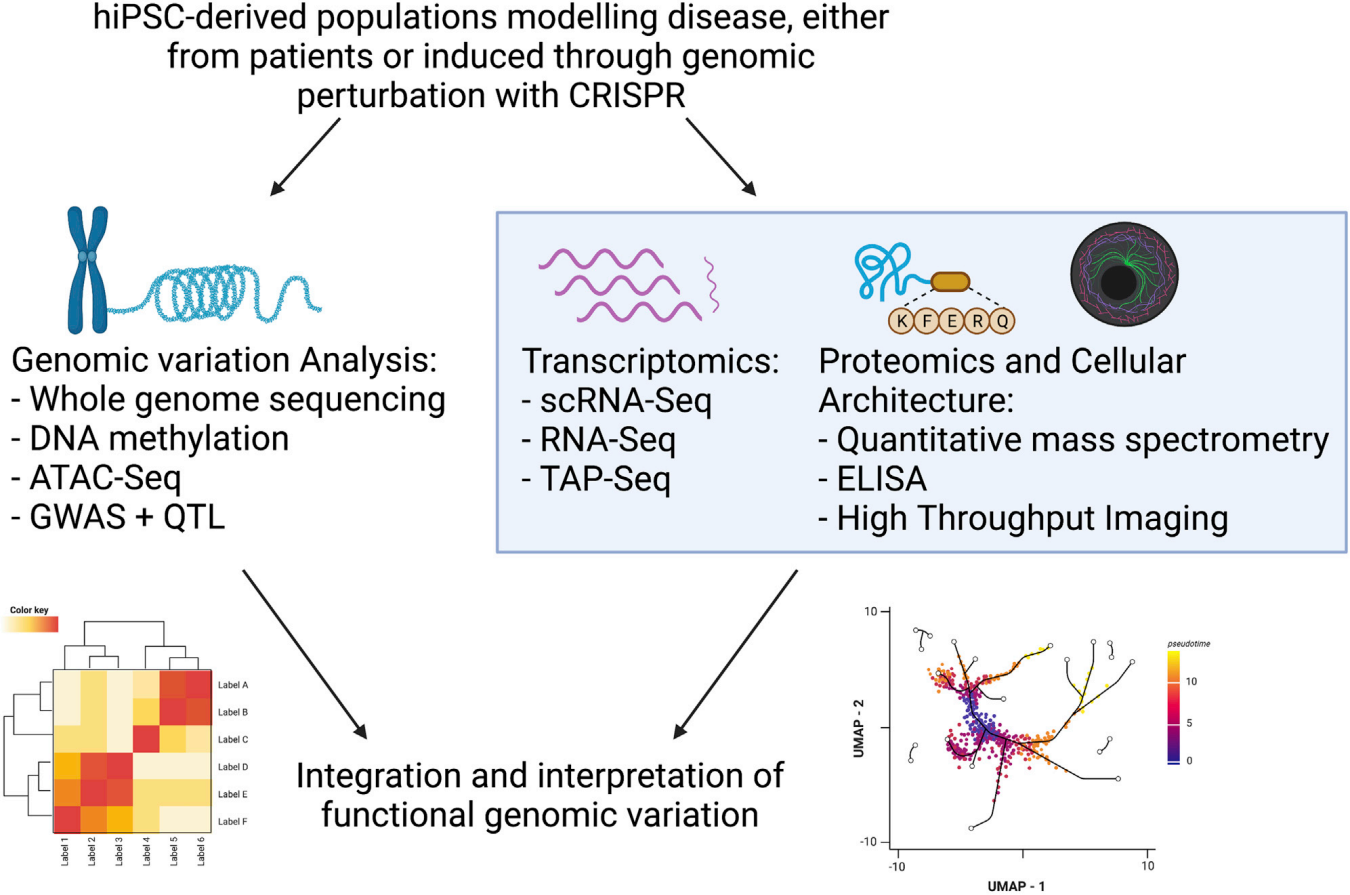
Workflow of hiPSC-based disease modeling and downstream multi-omics analysis hiPSC-derived control and disease-specific cell lines are generated either from healthy individuals, patients, or via CRISPR-Cas9 editing. Genomics, transcriptomics, and/or proteomics analyses are performed for each experimental condition. Conditions are cross-compared and interpreted to elucidate disease mechanisms and identify potential therapeutic targets. Created with Biorender.com.

Ever since the human genome was sequenced, there has been strong interest in unraveling the complex interactions between genotype and phenotype through population-wide studies using genome-wide association studies (GWASs), as well as whole-genome sequencing (WGS) or whole-exome sequencing (WES). Such approaches, while useful in identifying potential disease-related loci and SNPs, lack the capacity to interrogate the molecular basis of diseases (Visscher et al., 2012). The rise of multi-omics technologies has enabled unbiased characterization of biological systems. Combining these technologies, functional genomics identifies the relationship between genomics and phenotypic mechanisms (Figure 1).

In this review, we discuss the use of functional genomics in exploring genetic variation, advancing hiPSC-based disease modeling, and in drug discovery. First, we examine how hiPSC technology can be used to investigate rare disease variants. Then we discuss how functional genomics can improve 2D and 3D *in vitro* models of hiPSCs for monogenic and complex diseases. Finally, we explore the value of functional genomics in facilitating drug screens and developing personalized medicines.

### Uncovering disease-linked genetic variants in hiPSCs

Genetic variants, including single-nucleotide variants (SNVs) and copy number variants (CNVs), in the coding and non-coding regions of the human genome can play an important role in human traits and complex diseases. Discovering the mechanistic interplay through which these variants are associated with pathological states by high-throughput methods (metabolomic, transcriptomic, and proteomic) is one of the main objectives of functional genomics (Bonder et al., 2021).

Historically, hiPSCs have mostly been used to model highly penetrant genetic variants, which lead to substantial phenotypic effects (Itzhaki et al., 2011; Lee et al., 2009; Liu et al., 2011). Nevertheless, the effects of common genetic variants, which induce moderate phenotypical changes, is also a growing area of research, as it can provide key insights into drivers of complex diseases (Warren et al., 2017). Because of the small effect size of common disease-associated risk alleles, a major limitation in the use of hiPSCs to expose subtle effects of genetic variants is posed by the lack of sufficiently powered genomic tools (Nazor et al., 2012; Soldner et al., 2016). Thus, coupling the use of large-scale cohorts of hiPSCs to functional genomic studies offers the unique opportunity to investigate how common disease loci and rare genetic variants can contribute to cell state in both a physiological and pathological context (Table 1).

**Table 1:** Considerations when using iPSCs for functional genomics studies

	Current recommendations	Limitations
hiPSC differentiation and maturation for omics measurements	careful consideration of the disease/phenotype to be modeled; e.g., modeling chronic aging-related diseases or multi-systemic diseases to improve efficiency of hiPSC differentiation: forward programming, optimization of culture media to improve homogeneity of organoids: by bioprinting or automation	multilineage directed differentiation protocol not well established, time as limiting factor, variable efficacy depending on hiPSC lines forward programming relies on manipulations, such as transgene overexpression, chemical treatments, that can induce stress and promote unexpected alterations in genome, epigenome, and phenotypes 3D modeling may add complexity and variability, and reduce scalability automation can be expensive bioprinting is of limited access
Individual or pooled genotype study design	pooled genotype study design in cell villages is suitable for associating phenotypes to natural variation in population because of variability in differentiation efficacy of hiPSC lines, in pooled study the genotypes present in the initial cell population need to be reassessed after differentiation and before any perturbations. This prevents underrepresentation of genetic variants due to low differentiation efficacy in individual genotype studies, it is recommended to incorporate multiple disease lines to improve statistical power; add isogenic control lines	pooled design does not allow study of non-cell-autonomous mechanisms isogenic control lines may harbor unexpected off-target edits that affect the phenotypes of interest
Choice of perturbation technologies	CRISPR-based perturbation is recommended due to its adaptability to gain- and loss-of-function approaches. Genome-wide guide RNA libraries are readily available for pooled CRISPR screens; sgRNA can also acts as a barcode and simple readout for enrichment after phenotypic selection PROTACs as alternative choice for knockdown of lethal genes at the protein level	limited selection of existing validated PROTACs; genome-wide library is not yet available

Seminal studies have focused on the generation of large-scale, well-characterized hiPSC libraries from healthy donors, incorporating accompanying genomic and phenotypic data (Carcamo-Orive et al., 2017; Kilpinen et al., 2017). Comprehensive analysis of the data obtained from those libraries demonstrates key detectable effects of common genetic variance at all phenotypic levels, including effects on the epigenome, transcriptome, and proteome (Kilpinen et al., 2017). Mapping of expression quantitative trait loci (eQTL), which are regions in the genome that harbor polymorphisms associated with changes in gene expression levels, is a common approach to link the effect of genetic background on mRNA expression. In a study by Kilpinen et al. (2017), the systematic generation, genotyping, and phenotyping of 711 hiPSC lines from 301 healthy individuals identified hiPSC-specific eQTLs, which tag loci associated with disease. For instance, an eQTL regulating germline telomerase reverse transcriptase (TERT) expression is suggested to regulate telomerase activity in a genotype-dependent manner, indicating distinct cancer susceptibility among different hiPSC lines.

In a more recent study, Bonder et al. (2021) further scaled up this population-wide approach by integrating data from 1,367 hiPSC lines obtained from five previous studies (Banovich et al., 2018; Carcamo-Orive et al., 2017; Kilpinen et al., 2017; Panopoulos et al., 2017; Pashos et al., 2017) to establish the relationship between genotype and RNA sequencing (RNA-seq) data. This study revealed 21,548 genes whose expression levels are associated with genetic variants (eGenes), including eQTLs that mapped on exons, splicing regions, and alternative polyadenylation. Out of the eGenes detected, 995 had never been described from previous studies and mapped to loci that are linked to cancer and embryo development. Furthermore, analysis of hiPSCs derived from patients affected by rare diseases highlighted enrichment for expression outliers, indicating the importance of the study in prioritizing genes that have a causal mechanism in disease pathogenesis. Last, comparison between eQTLs and previously known GWAS hits identified 836 colocalization events that are specific to hiPSCs. Overall, this work represents a novel approach to generate reference datasets that will be pivotal in the identification of rare variants.

By coupling functional genomics tools with hiPSC differentiation protocols, several studies have identified genetic variants that can affect gene expression in specific contexts without being associated with steady-state gene expression. These include developmental stages (Cuomo et al., 2020; Jerber et al., 2021); exposure to an environmental stimulus, such as hormones, drugs, and vitamins (Findley et al., 2021); and disease contexts, including cardiomyopathy (Ward et al., 2021), myeloid leukemia (Wang et al., 2021b), and autism (Cederquist et al., 2020). In an effort to understand regulatory effects of genetic variation on cell stress during cardiomyopathy, Ward et al. (2021) developed an *in vitro* model of hypoxia based on hiPSC-cardiomyocytes derived from 15 genotyped patients. Integrating gene expression data with DNA methylation and chromatin architecture, they identified novel eQTLs and described their link to complex traits in a disease-mimicking setting as well to genetic responses under hypoxia-induced cell stress (Ward et al., 2021).

Continuous efforts in banking large amounts of high-quality, thoroughly characterized hiPSCs have allowed population-scale studies to increase steadily in sample size and frequency (Mitchell et al., 2020). Nevertheless, such studies are still greatly hampered by the requirement of culturing hundreds to thousands of donor lines, which is expensive and time consuming. To improve scalability and further minimize experimental noise, novel computational algorithms have been established to allow the culture of cells from several unrelated donors together as “villages” in a single dish (Mitchell et al., 2020). Mitchell et al. (2020) describe the use of Census-seq to associate cellular phenotypes to each individual donor genotype, while other computational demultiplexing approaches use single-cell RNA-seq (scRNA-seq) reads to assign each cell to an hiPSC line in the pool (Huang et al., 2019; Kang et al., 2018; Xu et al., 2019). Powell co-workers have demonstrated that the inter-line variation in gene expression is not altered by the experimental conditions when comparing hiPSC lines cultured as villages or separately (Neavin et al., 2021). Thus, researchers can recognize pool effects and differences between hiPSC lines not previously documented.

Capturing developmental processes involved in cell maturation requires long-term hiPSC differentiation, and most multiplexed studies, including the village approach, are restricted to a shorter time frame. To tackle this issue, Jerber et al. (2021) used a multiplexed strategy to map midbrain neuronal development and maturation through the differentiation of 215 hiPSC lines from the HipSci collection. Together, their findings show that pooled differentiation of hiPSCs coupled with scRNA-seq enables mechanistic studies of genetic variants and fates during several developmental stages and diseases (Jerber et al., 2021). Although promising, such population-scale, pooled experimental design might fail to accurately identify and measure the effects of non-cell-autonomous traits. Furthermore, it is not yet fully clear whether the interaction between cells obtained from different donors might alter specific cellular networks. Therefore, care should be taken when investigating biological processes known to heavily rely on cell-cell communication (Table 1).

Overall, hiPSC differentiation can be an invaluable tool to identify the molecular targets of non-coding or disease-linked genetic variants, providing insights into disease modeling and therapeutic discovery.

### Modeling monogenic diseases

Pathogenic mutations in one specific gene of the entire human genome can lead to monogenic disorders (Chen et al., 2020). Over 10,000 monogenic diseases are known (Chen et al., 2020). Examples include cystic fibrosis, Rett syndrome, Huntington’s disease (HD), monogenic diabetes, and polycystic kidney disease. The combination of functional genomics and hiPSCs can shed light on disease mechanisms that are difficult to model in *in vivo* systems and provide information on possible pathological mechanisms and key controllers of cell fate. For example, Mehta and colleagues have pioneered the use of 2D culture of hiPSC-derived cortical neurons from patients with juvenile-onset HD to investigate the effect of HD on cortical neurons (Mehta et al., 2018). Delays in corticogenesis have previously been implicated in HD, but striatal neurons remain the predominant cell type investigated in HD studies (Mehta et al., 2018). By combining transcriptomic analysis, electrophysiology, and morphological measurements of neurites, the authors discovered a slower functional maturation of HD hiPSC-derived cortical neurons, which could contribute to disease etiology (Mehta et al., 2018).

Monogenic diabetes appears in multiple forms, with maturity-onset diabetes of the young (MODY) being most prevalent (El-Khairi et al., 2021). In this context, murine models often fail to recapitulate defects in β cell function, including lack of insulin secretion (El-Khairi et al., 2021). MODY is frequently associated with mutations in transcription factors, including HNF1A (MODY 3), HNF4A (MODY1), and HNF1B (MODY5) (El-Khairi et al., 2021). Multiple mutations in the HNF1B gene are associated with the development of MODY5, including whole-gene deletions. Patients with a whole-gene deletion have the same clinical presentation as those with point mutations in the HNF1B gene (El-Khairi et al., 2021), suggesting haploinsufficiency. To investigate the effects of gene-dosage, El-Khairi et al. (2021) characterized isogenic HNF1B mutant hiPSC lines generated using CRISPR-Cas9 editing. They found that homozygous deletion of HNF1B hampers the ability of hiPSC to differentiate *in vitro* into pancreatic progenitors, while heterozygous deletion results in a reduced number of functional β-like cells compared with the wild-type counterparts (El-Khairi et al., 2021). RNA-seq shed light on the genes that are differentially regulated in response to homozygous or heterozygous knockdown and might underlie HNF1B-associated diabetes onset in humans (El-Khairi et al., 2021). Together these findings are consistent with a model in which HNF1B haploinsufficiency *in vivo* in patients might lead to reduced β cell numbers at birth and increased diabetes susceptibility later in life.

Recent investigations into the role of TBX5 in Holt Oram syndrome (HOS) have presented another example of the power of functional genomics with hiPSCs to identify gene interactions causing disease phenotypes *in vivo*. HOS is a rare monogenic condition (1:100,000) causing limb and cardiac abnormalities, including ventricular defects, due to loss-of-function mutations in the TBX5 gene that lead to haploinsufficiency (Kathiriya et al., 2021). scRNA-seq of hiPSC mutants for *TBX5* enabled the construction of gene regulatory networks (GRNs) linked to congenital heart disease (Kathiriya et al., 2021). GRNs showed that TBX5 dosage is critical for maintaining cardiac network stability and pointed out potential genetic interactions disrupted in TBX5-dependent congenital heart defects (CHDs), such as with MEF2C. Thus, by modeling hiPSC-derived cell types *in vitro*, one can understand their potential contribution to disease states. This reductionist approach can be extremely beneficial as it permits rapid, applicable, accurate, and in-depth analysis of many aspects of disease.

In addition to 2D modeling approaches, the past decade has seen the growth of 3D technologies. This involves the generation of miniature organ-like structures *in vitro*, which are now termed organoids (Hofer and Lutolf, 2021). While traditional 2D cultures provide high scalability and replicability, they restrict cell differentiation and fail to closely resemble *in vivo* tissue structures. 3D organoid cultures, on the other hand, support cell differentiation and recapitulate the cell-type diversity as well as morphological and functional features of *in vivo* organs (Langhans, 2018). Additionally, mechanical properties, such as matrix stiffness or gel degradability, can be adjusted in these 3D models to better mimic *in vivo* cellular behavior and structure (Jowett et al., 2021). Because of these advantages, organoids are increasingly used to elucidate the role of the microenvironment and cell-cell interaction in diseases. For example, fragile X syndrome (FXS), the most common monogenic cause of autism spectrum disorder (ASD), is due to CGG trinucleotide repeat expansion in the FMR1 gene (Kang et al., 2021). FXS forebrain organoids established from patient-derived hiPSCs have provided insights into the underlying neurodevelopmental abnormalities that were not apparent in murine models of the disease (Kang et al., 2021). This might help explain why drugs that seem promising in *in vivo* murine models of disease fail at clinical trial stage and could assist in future drug screens.

Functional genomics of hiPSCs can also be combined with novel technologies to provide new insights into disease mechanisms (Tables 2 and 3). For example, parallel translating ribosome affinity purification sequencing (TRAP-seq) combined with RNA-seq has been used to investigate mRNA ribosomal engagement during human development (Rodrigues et al., 2020). As mRNA translation is affected in Rett syndrome, it has been possible to assess the functional impact of mRNA interaction with the ribosome in the disease context (Rodrigues et al., 2020).

**Table 2:** Perturbation technologies commonly used in functional genomics

No	Technologies	Level	Types of perturbation	References
1	CRISPR technologies	DNA	based on CRISPR-Cas9 and its variants • CRISPR knockout: gene deletion by Cas9 nucleases • CRISPRi: reduction of gene expression using dCas9 • CRISPRa: upregulation of gene expression using engineered dCas9 fused with transcriptional activator • gene knockins: inserting gene into the genome	Deneault et al. (2018), Liu et al. (2020), Ross et al. (2020), Smargon et al. (2020), Tian et al. (2021)
		RNA	based on CRISPR-Cas13 • RNA degradation • RNA base conversion	Xu et al. (2021)
		epigenome	based on engineered dCas9 fused with KRAB repressive domain, DNMT3A, and DNMT3L • chromatin editing	Nakamura et al. (2021)
2	overexpression technologies	DNA	based on the delivery of foreign gene into target cells • transient transfection: episomal transgene expression • stable transfection: transgene integration into the host genome	Prelich (2012)
3	RNAi	RNA	based on RNA-dependent gene silencing mechanism induced by short RNA molecules • knockdown of mRNA level	Mohr et al. (2010), Wang et al. (2020)
4	PROTAC	protein	based on small molecules designed to degrade target proteins by ubiquitination and proteasomal degradation • protein knockdown: reversible reduction of target protein level, suitable for studying embryonic-lethal genes	Gao et al. (2020), Sun et al. (2019)

**Table 3:** Measurement omics approaches commonly used in functional genomics

Level	Technologies	References
Epigenome	ATAC-seq, ChIP-seq	Lopes et al. (2021), Martone et al. (2020)
3D chromatin organization	Hi-C	Ahmed et al. (2021), Lopes et al. (2021)
Transcriptome	RNA-seq (bulk or single cell)	Cuomo et al. (2020), Jerber et al. (2021), Park et al. (2021)
Proteome	ELISA, mass spectrometry, NMR	Park et al. (2021), Yates (2019)
Metabolome	mass spectrometry	Jalota et al. (2020), Zhao et al. (2018)
Optical phenotype	high-content imaging, flow cytometry, mass cytometry	Park et al. (2021)

ATAC-seq, assay for transposase-accessible chromatin with sequencing; ChIP-seq, chromatin immunoprecipitation sequencing; Hi-C, chromatin conformation capture sequencing.

Key to the advancement of our knowledge of monogenic disorders is a deeper understanding of how genotype affects clinical phenotype. While monogenic disorders are caused by mutations in one gene, the chromosomal location, penetrance, and variable expressivity of a given “pathogenic” mutation can vary widely (Goodrich et al., 2021; Shteinberg et al., 2021). In addition, genetic variation within families, genetic background, sex, and ancestry-related features may contribute to heterogeneous clinical manifestation of the disease (Volpato and Webber, 2020). Indeed, it has been reported that heterogeneity in hiPSC phenotypes is predominantly due to the genetic background of the donor rather than to non-genetic factors (e.g., passage, culture conditions) (Ho et al., 2021; Kilpinen et al., 2017). Furthermore, hiPSC-specific eQTLs highlighted that hiPSCs can significantly differ from their source cells in several GRNs (DeBoever et al., 2017; Kilpinen et al., 2017). Cumulatively, inter-individual variation may have significant impact on various levels of cellular phenotypes and functions. Apart from the genetic background, the donor-specific epigenetics landscape that is retained after hiPSC reprogramming may also affect cell variability. For example, Polycomb repressive complex and associated targets have been shown to contribute significantly to the non-genetic variability seen within and across individuals (Carcamo-Orive et al., 2017).

**Figure 2:**
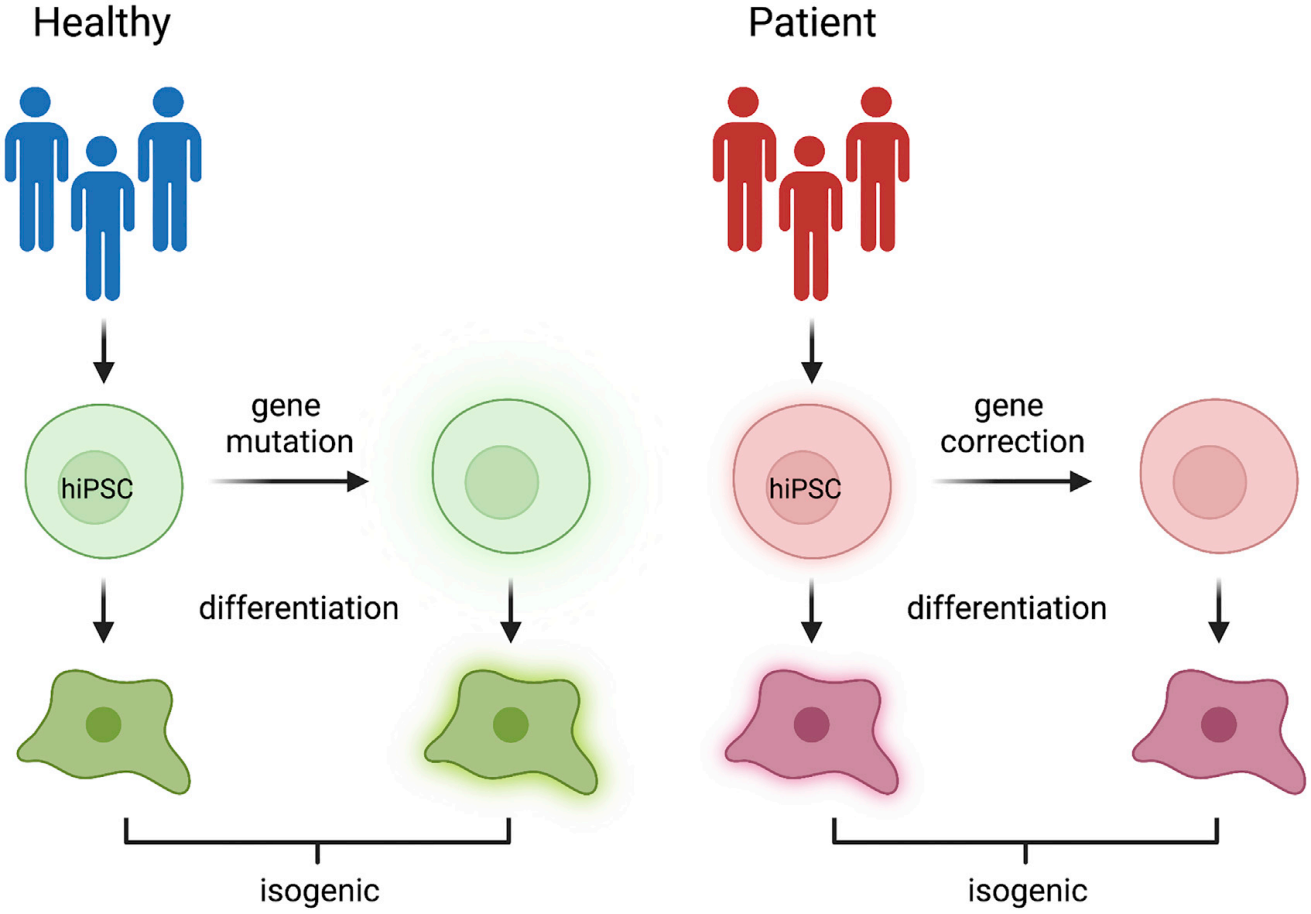
Generation of isogenic pairs of cell lines that differ by a single genetic modification Isogenic control cell lines can be created from healthy pluripotent stem cells to model the effect of a specific patient population. Alternatively, patient-derived hiPSCs can be corrected to serve as a genetic background control for *in vitro* disease modeling and drug screening. Created with BioRender.com.

As we strive to move toward more personalized models of disease, both specific mutations and genetic background should be considered in order to tailor an effective treatment to each patient. To this end, the creation of isogenic lines is key (Figure 2). Anastasaki et al. (2020) described the generation of multiple isogenic lines that harbor seven different neurofibromatosis 1-causing mutations engineered into one single male hiPSC line. The study showed different effects of the various neurofibromin mutations in 2D and 3D *in vitro* models. As the mutations are all engineered into the same isogenic cell line background, the effects observed are due to the individual mutations and not confounded by factors such as genetic background and sex (Anastasaki et al., 2020). As an alternative approach, patient hiPSC lines can be compared with gene-corrected isogenic control cell lines (Figure 2) (Sladen et al., 2021).

### Modeling complex diseases

In addition to monogenic disease, complex diseases that are affected by environment as well as genetic mutations can be modeled using hiPSCs and functional genomics. Recent functional genomics approaches have shed light on putative causal variants and uncovered molecular mechanisms underpinning complex cardiovascular diseases and neurological disorders in hiPSC-based disease models (Doss and Sachinidis, 2019).

Cardiovascular diseases are one of the leading causes of death globally in hospitals. However, the development of CHD is underexplored due to our limited ability to model the human heart *in vitro*. Recently, Lewis-Israeli et al. (2021) developed a high-throughput chambered heart organoid platform from hiPSC lines to study the consequences of pregestational diabetes (PGD) on heart development. With increasing recognition that the extracellular matrix (ECM) can affect cardiac cell state, researchers have also started seeding hiPSC-derived cardiac cells in solubilized ECM as well as synthetic hydrogels to better mimic physiological conditions (del Álamo et al., 2016) and developed new media formulations to support long-term cardiomyocyte maturation (Lewandowski et al., 2018). In another study, hiPSC-derived cardiac micro-tissues were printed using a micro-continuous optical printing system (μCOP). These 3D micro-tissues displayed tissue alignment and higher maturity than 2D cardiomyocytes, making them a promising system to recapitulate cardiac structures. The short time from design to printed tissue opens the possibility for the rapid generation of specialized and patient-specific complex disease models (Miller et al., 2021).

As in the case of the heart, the complexity of the human brain and the limited recapitulation of human conditions by animal models make it challenging to model neurological disorders. The high engineerability of hiPSC-based *in vitro* systems empowered by gene-editing tools has provided new opportunities to study brain biology and disorders (Table 2). Using CRISPR-Cas9 editing, hiPSC lines harboring mutations in ASD susceptibility genes (*CHD8*, *ASTN2*, and *AFF2/FMR2*) have been generated (Deneault et al., 2018; Wang et al., 2015). This allows researchers to examine the effects of single gene alterations on disease progression or prevention. Multimodal CRISPR interference (CRISPRi) genetic screens have helped to identify genes that are essential for neuronal survival and differentiation in healthy hiPSC-derived neurons as well as to modulate the expression of non-coding regulatory variants to drive ASD-associated phenotypes (Ross et al., 2020).

AD is a multi-factorial disease caused by dysregulation in various processes, such as trafficking, immunity, and lipid metabolism (Karch and Goate, 2015; Kunkle et al., 2019). It involves diverse risk factors and multi-step pathogenic processes, representing a challenge for disease modeling and drug screening. Through *in silico* analysis of multi-omics data, Wang and colleagues identified ATP6V1A as a key driver of late-onset AD (LOAD) (Wang et al., 2021a). To confirm its role in altering neuronal activity, CRISPRi was utilized in an hiPSC-derived NGN2-neuron (iN) model, which efficiently repressed the neuronal expression of ATP6V1A. ATP6V1A-deficient iNs demonstrated significantly reduced neuronal activity, which was further impaired upon Aβ42 exposure. The successful recapitulation of LOAD-related neuronal pathologies highlights the potential of the ATP6V1A-deficient hiPSC-derived NGN2-neuron model as a promising *in vitro* system. CRISPRi can be applied to precisely modulate the expression of other neurodegenerative LOAD-related genes, like SNCA, MAPT, and APP, as well in hiPSC-derived neurons (Heman-Ackah et al., 2016).

The apolipoprotein E (APOE) polymorphism ApoE4 is a major risk factor for sporadic AD (Lin et al., 2018; Park et al., 2021). Murine models are less favored in the study of AD pathology, as mouse APOE shares low homology with human APOE. Thus, to create a disease model that better mimics the neuropathological hallmarks of early-onset AD *in vitro*, an isogenic hiPSC line harboring the polymorphism ApoE4 has been generated through CRISPR-Cas9 modification (Lin et al., 2018; Park et al., 2021). Nevertheless, hiPSC models do have limitations, such as incomplete recapitulation of features of mature physiological neurons.

CRISPR activation (CRISPRa) has been leveraged to stimulate the neuronal differentiation of hiPSC for disease modeling and drug screening (Li et al., 2017). CRISPRa screening machinery also has the power to identify genes that modulate human neuronal survival or oxidative stress and provides biological insights that complement CRISPRi screens. For example, Tian and colleagues discovered through combinatorial CRISPRa/i screens that prosaposin (*PSAP*) deficiency could result in redox imbalance and neuronal ferroptosis linked to neurodegenerative disease (Tian et al., 2021). Apart from inducing disease phenotypes, CRISPR can be used to identify and validate plausible drug targets. For example, the knockdown of the *DSCAM* gene, which is overexpressed in Down syndrome (DS) patients, rescued diminished DSCAM/PAK1 signaling in a DS hiPSC-derived cerebral organoid model, which in turn restored neuronal proliferation and reversed impaired neurogenesis (Tang et al., 2021). In another example, a CRISPRi screen of over 5,000 long non-coding RNA (lncRNA) loci identified *lncGRS-1* as a potential lncRNA therapeutic target in malignant glioma (Liu et al., 2020). These results highlight the significance of CRISPR-based hiPSC-derived disease models in mining therapeutic targets to treat complex neurological conditions.

Functional genomics has also been employed for modeling non-neurological complex diseases in hiPSC-based systems, such as cancer. For example, a severe congenital neutropenia (CN) iPSC model carrying a RUNX1 mutation associated with leukemia effectively recapitulates leukemogenesis in CN (Dannenmann et al., 2021). This *in vitro* model system is particularly important as there are currently no animal models that can recapitulate the *in vivo* stepwise CN transition to acute myeloid leukemia (AML). In addition an hiPSC-based clonal evolution model of AML has been established through sequential induction of three driver mutations in AML via CRISPR-Cas9 technologies (Wang et al., 2021b). This leukemogenesis model better represents the founding clone of AML, shedding light on mutations that can arise during early AML and its progression. Similarly, known driver mutations of glioblastoma (GBM) have been introduced into hiPSCs, generating levels of intra- and inter-tumor heterogeneity similar to those seen in patient-derived GBM models (Koga et al., 2020). These represent very useful models for drug repositioning as well as for developing a broad range GBM drugs of low-resistance potential.

### Functional genomics in hiPSC-based drug screening

hiPSC-based disease models that accurately recapitulate disease physiology represent invaluable tools for phenotypic screening to identify druggable targets for therapeutic intervention and screen for candidate drugs. Indeed, all five approved first-in-class drugs for neurodegenerative diseases discovered in the last 20 years were developed through phenotypic screening (Swalley, 2020), likely because conventional target-based screens poorly represent multi-factorial complex diseases. However, even phenotypic-based endpoints are limited by the fact that an abnormal phenotype could be the result of distinct underlying pathophysiological genetic networks.

In that regard, functional genomic-based screening, which captures large amounts of biological data through multi-omics measurements, could improve the success of developing drugs for diseases with a complex genetic component. In a recent study, functional genomics combined with hiPSC-based models has been used to map GRN dysregulated in heart valve disease (Theodoris et al., 2021) and screen for drugs that “correct” the dysregulated GRN. This screening approach is unusual, because therapeutic hits were identified based on the modulation of core regulatory elements underpinning the disease, rather than on downstream effectors of the disease phenotypes (Theodoris et al., 2021).

A network-based screening platform could be applied to any complex disease with a genetic component. For example, in AD (Karch and Goate, 2015) the identification of disease-modifying targets has been highly inefficient when using conventional approaches (Raja et al., 2016). Leveraging the large amount of omics data from 1,300 hiPSC-derived human cerebral organoids, Park et al. (2021) validated an AD network-based model constructed from existing studies, and performed *in silico* perturbation analysis on the AD network model to screen for candidate molecules from US Food and Drug Administration (FDA)-approved drugs. Candidate drugs were tested using a high-content screening platform to assess their therapeutic efficacy. This serves as a good example of how the synergistic combination of functional genomics and hiPSC-based disease models can capture the underlying dysregulated GRN of a disease, leading to improved target discovery and drug screening.

### Limitations of the study

Despite the many advantages of hiPSCs for disease modeling, there are some limitations and challenges that need to be overcome. One major drawback is the limited maturation of iPSCs into functional adult cell types within a reasonable time frame (Subramanian et al., 2019), as the differentiated cells often display fetal-like characteristics (Subramanian et al., 2019). Indeed, functional genomics can be used to define and improve the maturation status of hiPSC-derived cell types. Recently, Kannan et al. (2021) combined computational modeling with functional genomics to develop a new benchmarking tool that can be applied to multiple cell types and across species. They propose a scoring system, called transcriptomic entropy, to assign hiPSC-derived cardiomyocytes into maturation categories based on gene expression (Kannan et al., 2021). The entropy score was also applied as a pseudotime metric and validated using *in vivo* cross-species studies (Kannan et al., 2021). Similarly, Subramanian et al. (2019) have developed an analysis pipeline using scRNA-seq to evaluate the cellular composition of kidney organoids developed from patient-derived hiPSCs and benchmarked them against fetal and adult human kidneys.

The pathogenesis of numerous diseases involves cellular crosstalk, often among different organ systems. For example, AD pathology is defined by neuronal and microglia crosstalk, and, by using functional genomics tools on neuronal and microglia co-cultures differentiated from patient-derived hiPSCs, new drug targets might be discovered (Li et al., 2017; Tian et al., 2021). There is a need, therefore, for reproducible, multi-cellular *in vitro* models that can capture this complex array of interactions to better understand and find treatments for diseases. However, concurrent differentiation of multiple lineages can still be technically challenging, in terms of defining appropriate culture conditions and unifying the timeline of differentiation protocols (Table 1). While the organoid systems offer the multicellularity, they suffer from substantial variability in formation efficiency, end-point morphology, and function, which is due to the stochastic nature of *in vitro* self-organization (Table 1). Reducing this variability will be essential to fully capitalize on the potential of organoids in disease modeling, drug screening, and regenerative medicine (Hofer and Lutolf, 2021). In this regard, bioprinting technology has been successfully applied for the generation of kidney organoids with highly reproducible cell number and viability (Lawlor et al., 2021). In the future, such an approach might replace current manual protocol production for organoids (Hofer and Lutolf, 2021; Lawlor et al., 2021). Robotic technology is also contributing to the development of fully automated high-throughput workflow to generate organoids. For instance, an automated liquid handling system has enabled high-throughput-compatible production of brain organoids with homogeneous cellular composition (Renner et al., 2020). Finally, methods for high-throughput phenotyping and scoring cellular heterogeneity in organoids will also be beneficial, in particular, in the context of drug screening platforms (Sharick et al., 2020).

hiPSC-based disease modeling will continue to benefit from rapid advances in gene targeting. For instance, a new way of introducing perturbations of genes in tandem via multiplexed single guide RNAs (sgRNAs) (Table 2) allows insights into combinatorial regulation of different genomic regions. This has proved particularly valuable in analyzing enhancer regions (Carleton et al., 2017; McCarty et al., 2020; Yan et al., 2021). Additionally, inducible, transcription factor-mediated forward programming approaches are increasingly implemented for boosting the efficiency of hiPSC differentiation toward specific cell types (Lange et al., 2020). Forward programming coupled to extensive phenotypic analyses will also provide a platform for identification of GRNs and enhance our understanding of differentiation.

Loss-of-function gene perturbation can be lethal and, thus, difficult to study. To overcome this issue, gene expression can be knocked down transiently through RNAi or CRISPRi; however, knockdown duration and efficiency are difficult to control (Table 2). An alternative might be the use of protein-level knockdown strategies, such as proteolysis-targeting chimeras (PROTACs) technology, which is based on bifunctional small molecules designed to knock down target proteins by ubiquitination and proteasomal degradation (Gao et al., 2020). PROTACs provide temporal control, allowing the knockdown of a target protein at specific time points and enabling the fast recovery of the target protein upon drug withdrawal (Sun et al., 2019), making it suitable for studying essential or lethal genes. Advances in measuring biological systems also accelerate our ability to fully characterize the omics beyond genomics and transcriptomics. New multiplexed mass spectrometry of individual ions (I^2^MS) can determine proteoforms, localized modifications on the proteins, as well as the denatured forms. In the future, comparing I^2^MS in disease genetic backgrounds could result in new insights as to the relationship between the genome and post-translational modifications of proteins (Yates, 2019).

In parallel with the application of new technologies, it is also important to tackle the challenges of big data analysis and data integration. Tarazona et al. (2021) highlighted some of the main challenges, such as sample size requirement to achieve high statistical power, signal-to-noise ratio within data, online storage and availability of linked datasets, and missing values skewing analyses. New workflows are being developed to overcome some of the issues, including targeted Perturb-sequencing (TAP-seq), which interrogates only a predefined panel of genes that are associated with pathways of interest (Schraivogel et al., 2020). This approach restricts the hypothesis space and tests needed for uncovering statistical significances, has increased sensitivity, and lowers sequencing requirements by 50-fold. At the same time, TAP-seq is platform independent and automatable, allowing large-scale screens (Schraivogel et al., 2020).

Finally, many studies are still based on the use of single cell lines (Cruz et al., 2017). Currently, there is debate as to whether any statistical analysis using biological replicates from one cell line is appropriate, or whether these replicates are subsamples or pseudoreplicates, resulting in incorrectly performed statistical tests with limited robustness or reproducibility. According to simulations, most single-cell studies are underpowered (Zimmerman et al., 2021). This is challenging as hiPSC lines from multiple relevant sources may be hard to acquire and/or expensive to grow, so transparency is needed from single-cell-type studies and conclusions are of limited value when additional cell lines are not available. When examining monogenic diseases, it may be beneficial to reproduce mutations with gene-editing or knockdown approaches in wild-type hiPSCs to increase sample size. Additionally, using appropriate statistical tests according to sample size and replicate hierarchies should be prioritized (Serdar et al., 2021; Tirrell et al., 2018). Nevertheless, for the ultimate goal of functional genomics, different independent hiPSC lines are needed toward the identification of disease mechanisms and therapeutic targets that are broadly applicable to multiple individuals.

### Concluding remarks

Functional genomics aims to characterize the relationship between genotype and phenotype, by perturbing, measuring, and comparing different biological systems at multi-omic levels. iPSC technology provides a powerful approach to elucidate disease biology and develop therapeutic interventions. High-throughput analyses should be employed routinely to characterize hiPSC lines and understand the long-term impacts of reprogramming effects. hiPSC-based functional genomics provides unique value for a holistic understanding of multi-factorial complex diseases and, consequently, tackling the challenges of translational research. It is expected to continue benefitting from the rapid development of multi-omics technologies as well as advancement in the generation of better hiPSC-based 2D and 3D models.

## Author contributions

I.R.B., C.M.G., C.K., C.S.K., S.S., and J.Z.X. wrote the manuscript. F.M.S. and F.M.W. edited the manuscript.

## Conflict of interests

The authors declare no competing interests.
